# Exploring Work-Related Experiences of Newly Hired Hospital Nurses in Ghana: A Qualitative Study

**DOI:** 10.1177/23779608241279911

**Published:** 2024-10-09

**Authors:** Ernest Darkwah, Francis Annor, Seth Oppong, Sylvia Hagan

**Affiliations:** 1 Department of Psychology, College of Humanities, 58835 University of Ghana, Legon, Ghana; 2 Directorate of Research, Innovation and Consultancy, 107841 University of Cape Coast, Cape Coast, Ghana; 3 Department of Psychology, Faculty of Social Science, 54547 University of Botswana, Gaborone, Botswana

**Keywords:** Nurses, well-being, occupational health, healthcare

## Abstract

**Introduction:**

Early work-related experiences of newly hired employees can have important implications for their work performance over time and their future health and well-being trajectories. In health care work, such outcomes may hold implications for the services delivered and the patients whose lives depend on them.

**Objective(s):**

This study was conducted to explore the work-related experiences of newly hired hospital nurses in Ghana.

**Methods:**

Using qualitative methods, thirty-six (36) newly hired nurses in three (3) hospitals in the Eastern Region of Ghana were purposively sampled and interviewed. Thematic network analyses were applied to the data collected.

**Results:**

Five main themes emerged which summarized the experiences of the nurses. The themes indicated that newly hired nurses experienced the nursing profession as stressful and yet fulfilling, different from their preentry expectations with multiple sources of stressors and resources. Religion and faith, family support, and a perceived lack of suitable alternative sources of income emerged as the main motivators of the intention to stay.

**Conclusion:**

The findings suggest a highly stressful and quite unpredictable work environment for newly hired nurses. Despite this, they are determined to stay on the job. Employee assistance programs that have specialized components targeted at providing emotional and psychosocial assistance to newly hired nurses are recommended.

## Introduction

Organizational researchers have long observed that employees’ experiences on the job and their subjective interpretations of these experiences can have important implications for their work outcomes. Such experiences may come from interactions with multiple factors both within and outside their occupational spaces (Mustika & Martdianty, 2023; Zhenjing et al., 2022). Within the workspace, employee experiences relating to factors such as interactions and relations with superiors and co-workers, work tasks, organizational clients, organizational resources, and structures may be impactful for both their health and occupational decision making (Adatara et al., 2018; Elbejjani et al., 2020; Mensah et al., 2024). For example, while Adatara et al. (2018) report that managerial interference and inadequate resources make the training and managing of nurses difficult for nursing managers in Ghana, Mensah et al. (2024) identified workplace bullying as a significant human relations problem that poses a threat to the mental health of Ghanaian nurses in general.

For newly hired employees, the initial on-the-job experiences can become even more important for the work attitudes they develop over time and their future work and health trajectories. Duchscher and Windey's (2018) Stages of Transition Model indicate that these initial experiences may include experiences of transition shock within the first year of employment and Labrague (2024) reports that recent nursing graduates undergo a period of reality shock within the first two years of starting their careers. Even though work attitudes may change over time (Konstantinou et al., 2017), the initial experiences can form a lens through which future work experiences may be interpreted. Thus, within healthcare systems, although the experiences of all workers are important, the experiences of newly hired nurses, in particular, are crucial as the outcomes associated with these experiences have direct implications not only for their own health and career decisions but also for the future of the health systems within which they work.

In the context of Ghana, reports of increasing attrition among newly hired nurses attributed to many factors including burnout and a desire to seek greener pastures abroad have emerged (Ghana News Agency, 2023, Nyande et al., 2024; Opoku et al., 2022; Poku et al., 2020). The threat of a shortage of human resources for health that this trend poses has forced national policymakers to begin to find retention strategies that not only help to stem the outflow of nurses but also ensure their health and well-being on the job (see Asamani et al., 2021). As part of these efforts, there is a need for qualitative humanistic inquiries that delve deep into newly hired nurses’ specific experiences on the job that can provide insight to policymakers and intervention strategies.

## Review of the Literature

Several studies note that on-the-job experiences and their subjective interpretations are linked to important work behaviors and outcomes including satisfaction, turnover intentions and turnover, citizenship behaviors, and counter-productive behaviors, among others (e.g., Ansari et al., 2013; Bhui et al., 2016; Chang & Smithikrai, 2010; Fernandez, 2008; Malek et al., 2018; Testa et al., 2020). Further, employees’ interpretations of their work environment have been linked to health and well-being outcomes including stress-coping, emotional exhaustion, and burnout (Brienza & Bobocel, 2017; Nikolova et al., 2017; Pignata et al., 2017).

This existing literature identifies healthcare work as one of the most complex and stressful jobs in which employees may obtain experiences that may be impactful for both their physical and mental health and well-being (Jordan et al., 2016; Rink et al., 2023). Nurses, in particular, are consistently exposed to many negative work experiences, including job-related stress, burnout, and physical and mental health problems (Elbejjani et al., 2020; Izdebski et al., 2023; Nam et al., 2016; Riley et al., 2017; Rink et al., 2023; Sharma et al., 2014; Tuxford & Bradley, 2015). The job of hospital nurses necessarily involves handling human suffering and displaying emotional labor (Ingebretsen & Sagbakken, 2016; Nam et al., 2016). Workers involved in such human service occupations often suffer higher levels of emotional exhaustion (Annor et al., 2023; Asiedu et al., 2018; Näring et al., 2012).

However, the Salutogenic Model of Health (Eriksson & Lindström, 2008) suggests that individual health outcomes in response to stressful circumstances depend on the identification and use of available coping resources (Hanson, 2007; Xanthopoulou et al., 2007). For example, nurses with longer tenure may have a better understanding of work demands, giving them a sense of control and resilience through adaptation due to longer exposure to the work situation. Thus, experienced nurses may develop mental preparedness for the work demands (Bakker et al., 2022; Demerouti & Bakker, 2011; Demerouti & Bakker, 2023). In contrast, new nurses often lack such exposure and, therefore, experience the work differently. There is evidence that new nurses are vulnerable to stress due to factors such as lack of experience, unfamiliarity with the work environment, and lack of personal resilience to respond to traumatic experiences on the job (e.g., Qiao et al., 2011). These vulnerabilities in new nurses have implications for their ability to make decisions on the job, their job performance and efficiency, and their mental health (Liang et al., 2018).

Yet, extant research on healthcare professionals has paid less attention to the experiences of new nurses on the job. Thus, research insights into the specific experiences of such new nurses seem inadequate in the existing literature. The limited research in this regard has been done mostly in developed countries with little attention to the experiences of nurses in sub-Saharan Africa. As noted by Asiedu et al. (2018, p. 612), “nursing practice in sub-Saharan African countries likely differ from developed countries due to structural differences in healthcare systems.” Faced with structure challenges such as high patient-to-nurse ratio and resource constraints, new nurses in developing countries such as Ghana are likely to face greater difficulty in adapting to the work environment. This study sought to contribute to efforts aimed at plugging this gap by exploring the lived experiences of newly hired nurses and developing in-depth insights into their work situations. The idea is that understanding these experiences could inform policy and programs on the training of nurses and their orientation on the job. The main aim of this study was to explore the work-related lived experiences of newly hired hospital nurses.

## Methods

### Design

The qualitative approach with interpretive phenomenological design was adopted for this study. This approach and design are best suited for studies that aim to investigate shared lived experiences of a phenomenon (Attride-Stirling, 2001; Moustakas, 1994; Van Manen, 1990). The researchers utilized this approach to generate in-depth insights into the newly hired nurses’ subjective meaning-making and interpretations of their own realities regarding their work situation in this study.

### Research Questions

To achieve the research objectives, the researchers sought to answer four research questions:

(1) How do newly hired nurses experience the health care work after employment?
(2) How do the experiences of newly hired nurses compare to their preentry expectations?
(3) What major stressors confront newly hired nurses on the job?
(4) How do newly hired nurses identify resources (if any) to help them manage the stressors on the job?

### Setting and Sample

The study was carried out in the Eastern Region of Ghana. Newly hired trained nurses were approached in three conveniently selected health facilities (i.e., one public hospital and two clinics) in the region and requested to voluntarily participate in the study. The hospital and clinic were in the regional capital, Koforidua.

Participants for this study were selected using purposive and convenient sampling techniques. Purposive sampling was employed to ensure that individuals who had recently been hired as hospital nurses in the Eastern Region of Ghana and had experience working in hospitals were included in the study. This method allowed for the selection of participants who could provide rich and diverse insights into the work-related experiences. Additionally, convenience sampling was utilized to recruit participants based on their availability and accessibility to the researchers, facilitating the recruitment process and enabling a timely collection of data.

Thirty-six (36) nurses meeting the inclusion criterion volunteered to participate and were selected. 

## Inclusion Criteria

In this study, “newly hired nurse” is defined as nurses with up to three years of experience who work in a hospital or clinic. To meet the “newly hired nurse” criterion to participate in the study, participants had to have been on the job for between one month and three years. The decision to include nurses with up to 3 years of experience was based on the recognition and empirical evidence that the transition period for newly hired nurses extends beyond the initial few months and may encompass up to 3 years as they adjust to the demands of their profession and work environment (see Duchscher & Windey, 2018; Labrague, 2024).

## Ethical Consideration

Ethical approval was obtained from the Departmental Research Ethics Committee (DREC) of the Department of Psychology, University of Ghana (DREC/017/19-20). Each participating nurse was given both a written and oral explanation of the study and its purpose. Each participant signed an informed consent form before participating. Participants were assured of confidentiality and anonymity of the information they provided; the data collected were stored in password-protected files accessible only to the first author. Specifically, the researchers utilized an online Dropbox account to securely store the data file.

## Data Collection

Data for the study was collected over two months (September, October, and 1st week of November 2020). The researchers utilized in-depth individual interviews to collect data from participants at times (off-work days or after-work hours) and places of their choosing. The data were collected in the hospital or clinic. In-depth interviews were used to obtain first-hand data from participants’ own narrations and descriptions of their experiences on the job. The interviews were conducted using a semistructured interview guide. The guide only contained the main interview questions based on the stated research questions, which were then probed further, based on a participant's specific responses.

Questions asked during the interviews included: (1) How have you experienced the health care work so far since you joined?, (2) Do your experiences so far match the expectations with which you joined this profession?, (3) What would you say are the main stressors confronting you on this job?, and (4) Are there any things, persons, factors or aspects of this work and work environment that you would say are resourceful to you and help you cope with the job?. These questions were reviewed by other qualitative researchers and piloted with three (3) nurses in the eastern region of Ghana.

Utilizing this interview approach allowed for a more focused exploration of the interviewees’ experiences and perspectives in relation to the research topic enabling the interviewer to probe deeper into relevant areas, uncovering nuanced insights that may not have been captured through predefined research questions alone. Also, by crafting interview questions based on the research objectives, the researchers ensured alignment between the data collected and the study's overarching goals. This helped to maintain coherence and relevance throughout the data collection process.

The lead author conducted the interviews with the participants. Data saturation was reached after interviewing 36 participants when novel information was absent in interviews. Thus, data repetition occurred reinforcing rather than enriching previously extracted information from participants and additional sampling did not yield new insights (Polit & Beck, 2017). Coupled with the data repetition, the decision to stop interviewing participants was also based on Creswell and Creswell, (2018) suggestion that interviewing 10 to 50 participants suffice to achieve data saturation in phenomenological studies (Creswell & Creswell, 2018).

The interviews lasted an average of one (1) hr and 23 min per session. For transcription and further analysis of the information provided, all interviews were audio-recorded with the written consent of each participant. The interview language was English for all participants. Three research assistants worked with us to carry out the transcription and coding. The research assistants were master's degree holders trained in conducting qualitative research methods in the social sciences.

### Data Analysis

The researchers used the COREQ 32 to guide the analysis process. Interviews were first transcribed verbatim, and each transcript was read thoroughly to get a sense of what had been said in the interviews. NVIVO v.10 software was then used to pick out codes from the transcripts. The exact words of the participants were initially coded using NVIVO v.10 software before basic themes were developed.

Attride-Stirling's (2001) Thematic Network Analyses (TNA) approach was then applied to further develop the codes into basic, organizing, and global themes. First, codes that seemed to convey the same idea were merged to become basic themes, and basic themes conveying similar experiences were further developed into larger organizing themes. The same procedure was followed to develop organizing themes into a larger, umbrella global theme that reflects the essence of the data collected and which also speaks to the main aim of the study. While conducting this research, the researchers ensured that their personal situatedness, experiences, and biases did not interfere with the data collection, analyses, and reporting in any way. The lead author who conducted the interviews has no experience in nursing and, therefore, left participants to share their experiences as they felt and interpreted them.

The data analysis was done with particular care for intercoder reliability where all authors discussed codes and themes and agreed on their representativeness of participants’ experiences before they were included. Any discrepancies or differences in coding were openly and thoroughly discussed. To resolve these discrepancies, a collaborative approach was employed where the research team held regular meetings to review and compare codes. Each discrepancy was examined in detail, and consensus was reached through discussion and re-evaluation of the data. Additionally, an iterative process was used, where adjustments were made to the coding framework based on team discussions, ensuring consistency and accuracy in the final coding. The data analysis process is presented in Table 1:

**Table 1.** Data Analyses Process Using Thematic Network Analysis Approach.

Codes	Basic themes	Organizing themes	Global theme
…I cry sometimes on the job …You feel you will run out of empathy …I go home feeling emotionally drained	High emotional content	Perceptions of nursing the nursing work	Work experiences of newly hired nurses
…you can save someone or kill them …you do your job well or someone could die …the responsibility for life or death is heavy	Life and death Profession		
… you are fulfilled after helping a patient …you get to learn the real value of life …it is definitely worth the stress	Fulfilling and worth the effort		
…You go home thinking about your patients … Sometimes you can see their faces …the shock of it all affects you mentally	Mentally distressing		
…it's way beyond the simulations in training …the reality is way beyond what I expected … I only thought about the salary	The reality is different from the expectations	Expectations versus realities	
…you think it's all nice until you enter …I thought it would be nice to be called nurse	Not as nice as it seems		
…the number of patients on you is unbelievable …You can process hundreds of patients a day …you get exhausted and make mistakes	High numbers of patients	Stressors on the job	
…because you are young, patients talk to you any how …some patients do not see you as competent “…some patients are very difficult to handle	Poor patient attitudes		
…some seniors think you know nothing …some of them are just bullies …the seniors use you for errands	Attitudes of senior nurses		
…prayer is the most important resource …God gave me this job, he will see me through …My faith in God keeps me going	Religion as a resource	Resources on the job	
…You obey your seniors and they will help you …if you are respectful to them, they like you in turn	Transactional friendships as a resource		
… my supervisor is amazing …my grandma calls me life saver	Supervisor & family support as a resource		
… I won’t have any income if I quit …what will I do if I quit?	Lack of alternative income	Motivators of intention to stay	
…I do it because it makes me feel important …you feel useful and worth something	A sense of worth		
…people respect you in your community …you are even recognized in church	Social prestige		

### Rigor

The principles of credibility, confirmability, dependability, and transferability were adhered to in this study. Credibility was ensured by promptly transcribing interviews on the same day. To ensure the applicability of findings across various contexts, the researchers involved nurses and midwives from different hospitals. Additionally, one external research assistant was engaged to validate the data's accuracy, relevance, and potential outcomes. All data, including interviews and field notes, were meticulously documented to ensure consistency and reliability. Participants were given access to verify transcribed files and quoted statements (Salvador et al., 2021). Finally, to ensure methodological rigor and transparency, this study follows the consolidated criteria for reporting qualitative research (COREQ) 32-item checklist (Tong et al., 2007)

#### Reflexivity

The interviews for this study were conducted by the lead author who had a PhD degree at the time of the study. His occupation during the study was an academic who is actively engaged in health promotion research in several workplace settings. The lead author had experience and training in qualitative research methodology which equipped him with the necessary skills to facilitate insightful discussions with the participants. Before the commencement of the study, efforts were made to establish a rapport with the participants to foster trust and openness during data collection. This involved initial interactions where the lead researcher introduced themselves, explained the purpose of the study, and listened to participants’ concerns or questions. Building rapport before the formal interview sessions helped create a comfortable environment for participants to share their experiences openly.

Participants were briefed on the lead researcher's background and motivations for conducting the study. This included the researcher's interest in understanding the unique challenges faced by newly hired hospital nurses in Ghana and their commitment to improving working conditions in healthcare settings. The lead researcher approached the study with a commitment to reflexivity, acknowledging personal biases and assumptions. Some of these biases included assumptions about the challenges faced by newly hired nurses based on existing literature. Additionally, the researcher's interest in exploring work-related experiences stemmed from a desire to contribute to the improvement of nursing practices and healthcare outcomes in Ghana. These characteristics may have influenced the dynamics of the interviews, potentially shaping the participants’ responses and the overall findings of the study.

## Results

### Sample Characteristics

The participating nurses comprised 34 Registered General Nurses and two (2) Midwife Nurses. Seventeen (17) were on rotation duties, 18 were staff nurses and one (1) was a staff midwife. Rotation nurses are fresh nursing graduates assigned to work in various healthcare facilities for one year across Ghana and this phase of their career is often referred to as their “national service.” Staff nurses, on the other hand, are full-time employees of the government. Thirteen (13) of the participants had worked for between one (1) month and one (1) year and 23 had worked for between one (1) and three (3) years. The minimum age among the participants was 18 and the maximum age was 38. There were 27 females and nine (9) males.

### Research Question Results

Overall, 15 basic themes of experience emerged from the codes that were generated from the data, and these further produced five (5) organizing themes. This findings section is organized along the five organizing themes.

### Perceptions of the Nursing Work

The specific ways in which participants perceived and experienced the nursing profession were wide, similar, and varied at the same time. The perceptions shared by the participants also paint a picture of the nursing profession as an emotionally charged, life and death, mentally distressing, stressful, and yet quite fulfilling and worthy profession. All participants described their experiences as being a kind of emotional roller-coaster. However, while some described this emotional aspect of the job as building their personality and making them stronger, others felt it left them drained. For instance, a staff nurse intimated that:

…Since I joined this profession, I have experienced things I never have before. I have seen death, I have seen suffering, I have seen people bravely embrace their end and yet, I have seen others who have bravely fought off death…it has made me stronger… (Staff nurse (SN) 3)

Another participant confirmed the emotional nature of the work but had a different interpretation

regarding its impact on her:

…. you can experience anger, joy, sympathy, sadness, disbelief, or just plain emptiness within one working day…It leaves you drained…so far, I am exhausted by the end of almost every work day… (SN2)

For other participants, the nursing profession brought with it a responsibility for the life and death of other human beings, which they considered heavy on them:

…I am on rotation currently, it means I go round all the departments to get a practical sense of what the job involves…in some places like emergency unit, someone's life practically can depend on you…you can help save a person or you can kill them by a simple mistake…it's a life and death profession…it's heavy… (Rotation nurse (RN) 8)

All participants (except for one) explained that the experiences on the job follow them home,

weighing on their minds and exposing them to mental distress:

…Sometimes you can see their faces when you close your eyes…those that frowned at you, those that smiled at you, those in pain, those in anguish…it can be mentally distressing…when I talk to the senior nurses, they say it will go…I just hope so… (RN 7)

Despite the seemingly negative experiences on the job, some participants were optimistic that things would get better:

…so far, it's been kind of a smooth and rough ride…somedays you really enjoy the job, some days you feel like going home and never coming back…but I have hope that as I keep doing this, things will smoothen up… (SN1)

### Entry Expectations Versus Realities on the Job

Participant narratives also shed some light on how they assumed the nursing profession would be before joining and how the realities on the job have been for them relative to what they thought it would be. Many of them agreed that the realities on the job did not match their entry

expectations:

…for me, it was my friend's sister…she was a nurse, wore this cute green uniform, earned a good income, and seemed very happy with her life…this was how I saw nursing…but since I joined…(laughs)…It's become clearer…this is not just about cute uniforms…it's much more… (RN 9).

For many of the participants, the motivation to join was from expectations of a decent, independent life:

…I joined this line of work because the pay is quite decent…as a woman, I felt it's a decent job that can give me a decent, independent life…and yes, it is…but I am also now realizing that I only thought about the salary, not what the actual work involves… (SN2)

Other participants felt that the training they received in training schools did not prepare them adequately for the realities of the job:

…In training school, they teach you to detach yourself, to not take things personal, you think you have learnt that, but when you get here, you realize that it is much harder to do that than you thought…the reality is way beyond what I expected… (RN 12)

### Stressors on the Job

Participants were also asked to share some experiences of stressors confronting them on the job. The idea was to find out what specific aspects of the job newly hired nurses felt were the most stressful to them. Key stressors identified included a high patient–nurse ratio, negative attitudes of senior nurses toward newly hired nurses as well as negative patient attitudes toward nurses in general and newly hired nurses in particular. For example, a staff nurse mentioned that:

…Our facility here is a big one, so we have a lot of patients on our hands, and it can get overwhelming sometimes…you can process hundreds of patients in a day…that is stressful… (SN 10)

The negative attitude of senior nurses toward newly hired ones was confirmed as a stressor in all the health facilities involved in the study:

…some of them are just bullies…in my first month, I nearly quit because of that…there was a time one of them told me I am not fit for the profession and the others were sitting there laughing…I felt so abused… (RN 8)

Some participants felt the bullying was a trend passed on from one cohort to the other:

…They show us no respect because they say the seniors, they came to meet showed them no respect…I guess they expect us to do same to those who will come after us…It's just a negative trend from cohort to cohort and it's really sad… (SN 16)

Patient attitudes were key stressors that almost all participants complained about. Some participants thought that patients sometimes had doubts about their maturity and competence as professional nurses:

…for me, so far, it has been some of the patients…it looks like they are used to seeing older, more matured nurses…and we the new ones just came out of school, so most of us are very young…they do not see you as competent… (RN 11)

### Resources on the Job

The experiences shared by the participants also shed light on what they considered to be resources for coping what the workplace stressors. In this regard, participants indicated in their accounts that some of their senior colleagues, their family, and most importantly, their religious faith were their key resources. A rotation midwife had this to say:

…the experience on this job will break you if you don’t have God on your side…when I am down, I pray and I feel alright… (Rotation midwife (RM) 1)

Although all participants mentioned their religious faith as a resource, for others, the support of their families is also something they fall on as a resource:

…my grandmother calls me life saver…when I come back from work, she wants to hear all about it every time…and she always gives me words of encouragements…she is such a resource… (RN 4)

Interestingly, participants also identified some of their senior colleagues as resources. However, the resourcefulness of the senior colleagues seemed to be transactional as it depended on what they gained from the newly hired nurses:

…if they send you on their personal errands and you go, they see you as respectful and they like you and they will support you…if not, you are pretty much on your own… (RN 3)

For another participant, his supervisor was the only exception:

…my supervisor is an exception, she is amazing…the rest, well, it depends…if you obey them, they like you…if you ask too many questions, they see you as disrespectful…so they can be resourceful if you just keep quiet and obey them… (RN 15)

#### Motivation to Stay

Participant narratives further gave some insights into their intention to stay on the job for a longer period. No participant said they intended to quit. A variety of factors were mentioned by participants as reasons why they intended to stay on the job. For example, a staff nurse commented that:

…what will I do if I quit?… this job pays better than other jobs like teaching or other civil service jobs…I can pay my bills and take care of myself…so yes, I intend to retire with this job… (SN 14)

The social recognition that seems to come with the job was also cited as a motivation to stay:

…people respect you in your community if you are a nurse, mothers want their daughters to follow your footsteps…it makes you feel important and respected…I enjoy that a lot… (SN 13)

In summary, it emerged that newly hired nurses experience nursing work in a variety of ways, some negative and others positive. The experiences shared have implications and provide some insights into opportunities for intervention strategizing and assistance programming in that context and contexts similar to the contexts of study. These implications have been discussed.

## Discussion

This study was conducted to explore how newly hired hospital nurses experience the nursing profession in the Ghanaian context using qualitative methods to generate insights into the sense-making processes of the nurses in relation to their work situation. The main recurrent themes that emerged create an impression of the nursing profession—in the eyes of the newly hired nurse—as a profession that is quite stressful with a significant impact on their mental health and yet worth doing.

The findings show that newly hired nurses experience the nursing work as emotionally demanding and one that places critical responsibility for the life and death of others on them. While some described this experience as having detrimental effects on their well-being, others had more positive interpretations, describing the experience as deepening their resilience on the job. The implication of this finding could be two-fold. The positive interpretation of this experience as helpful in personal development provides an opportunity for organizational leaders in this context to take steps that increase the capacity of newly hired nurses to continue to adopt positive outlooks for their job tasks. This could help them to be more effective on the job over time.

Previous studies indicate that employees’ subjective interpretations and evaluations of their job tasks can increase outcomes like work engagement, commitment, and acceptance where such interpretations and evaluations are positive (Faisaluddin et al., 2024; Fernández-Castro et al., 2017). However, the interpretation of the experience as emotionally exhausting provides evidence in support of the high prevalence of job-related emotional exhaustion among nurses (Dhar, 2013; Poku et al., 2020; Qian et al., 2024; Stab et al., 2016). While there is clear evidence that the experience of exhaustion cuts across all nursing groups and not only newly hired nurses, but the inexperience of newly hired nurses may also increase the impact on them relative to their experienced colleagues. This finding suggests employee assistance programs ought to target new nurses with psychosocial resources to deal with this kind of exhaustion. This is because previous studies provide evidence that job-related emotional exhaustion constitutes a significant threat to employee safety and health (Anger et al., 2024; Winkler et al., 2024). In the hospital setting, these negative outcomes can be consequential also for patients. Added to exhaustion, it becomes clear that newly hired nurses face considerable safety and well-being challenges on the job. Interventions with psychosocial support components may have to be developed to help newly hired nurses deal with exhaustion.

It also emerged in the study that, for all participants, the assumptions and expectations some of which led them to choose nursing as a profession did not match the realities of their experiences on the job. This dream-reality disconnect, as shown in the findings, has resulted in varying outcomes for the new nurses. Clearly, for some, with the benefit of hindsight, they would not have chosen hospital nursing as a profession, while for others it has led to a resilient resolve to face the job and succeed. This revelation alludes to the exposure of newly hired nurses to occupational reality shock, which is also common among nurses in several other contexts and is associated with a plethora of negative employee safety and health outcomes (Itomine, 2013; Kodama, 2017; Labrague, 2024). While the revelation is consistent with the evidence that occupational reality shock is often the main stressor faced by newly hired staff in organizations, it contradicts other findings that training received in medical and nursing schools often prevents newly trained nurses from experiencing such shocks (Bloom, 2019; Itomine, 2013).

Participants also narrated stressful job-related experiences in relation to the number of patients, patients’ attitudes toward nurses, and power dynamics between senior and junior nurses. While the overwhelming workload resulting from high patient numbers and, perhaps, the complaints about patient attitudes may be stressors confronting all nurses both junior and senior, the findings reveal specific dimensions of these stressors that seem to be particularly problematic to the newly hired nurses. It was revealed that perceptions of being new and young often make patients doubt their competence, contributing to poor patient attitudes toward them. Thus, there is still a need to closely monitor and support newly hired nurses to cushion them against such occupational stressors and build their human relations competencies to be better able to build rapport with patients and earn their trust. The human relations problems for newly hired nurses also had another, more serious twist to them: the attitudes of senior nurses toward newly hired nurses.

The problem of poor attitudes of senior nurses toward juniors and narratives of bullying behavior that was revealed as part of the main stressors in this work context has actually been identified in the existing literature as prevalent in the nursing occupation (Dellasega et al., 2014; Galanis et al., 2024; Park, 2012; Ribeiro & Sani, 2024; Simons, 2006; Vijayakumar & Rajagopal, 2024). This finding, therefore, adds to the growing evidence of this problem in the nursing profession globally and suggests, perhaps, the need for a concerted global effort aimed at developing guidelines to tackle it in this critical service work context. In the Ghanaian context, the finding provides more details by showing that even where junior nurses receive the needed support and comradeship from their seniors, the relationship is often transactional in that junior nurses must serve the personal needs of seniors in exchange for this relationship. Besides being a breach of general workplace ethics, it could create a vicious cycle of workplace bullying behavior as a normative practice.

The findings, however, show that in the face of these negative experiences on the job, newly hired nurses find strength and support mainly from their religious faith and their families. A sense of worth and social prestige associated with the nursing occupation emerged as complementary to faith and family support and seemed to mitigate turnover intentions. Particularly, religious faith seems to be a key resource that keeps the nurses going, despite the many negativities. Within the framework of the health promotion theory of Salutogenesis, this interpretation of faith in God as a sufficient resource to engage the stressors on the job indicates a sense of manageability which is a key ingredient in moving toward health, despite stress (Hanson, 2007). Additionally, previous studies demonstrate that job-related religious convictions can enhance one's sense of meaningfulness that can renew motivation and increase work engagement (Dik et al., 2024; Rothmann & Buys, 2011; Saks, 2011; Samul, 2024). Spiritual leadership and work engagement: a mediating role of spiritual well-being. With the findings showing that family support is already available for the newly hired nurses and a sense of self-worth and prestige gained from local community perceptions of nurses reinforce nurses’ determination to stay, increasing this sense of meaningfulness will be an additional support system and help buffer the nurses against the stressors.

## Strengths and Limitations

The study's strength lies in its ability to gather insights from different hospitals offering a diverse and comprehensive perspective. By using open-ended research questions, the researchers were able to collect rich and detailed data, allowing for a deeper understanding. Moreover, the trustworthiness of the study is strengthened by the participation of both female and male nurses across different age groups, ensuring a well-rounded representation of perspectives. However, since this qualitative investigation only involved two hospitals and a clinic in the Eastern region of Ghana, its findings are not generalizable. It is important to emphasize that the generalizability of findings was not the researchers’ main focus in this study, though some aspects of the findings may resonate with the experiences of newly hired nurses in other regions in other contexts. Additionally, the study is a dipstick study, and its findings can be complemented with findings from further larger studies that include more participants as well as views and experiences from senior nurses and institutional administrators. The inclusion of narratives from these other actors in that work environment could complement the information here and provide more contrastive and nuanced insights into the workplace realities in this work context.

## Implications for Practice

Healthcare organizations in Ghana can use the findings to inform their practices related to nurse recruitment, onboarding, and retention. Implementing structured mentorship programs and providing continuous support can help ease the transition for newly hired nurses and enhance their job satisfaction and performance. Policymakers in Ghana may consider incorporating the insights from this study into healthcare policies aimed at improving the nursing workforce. This could involve advocating for standardized onboarding processes, promoting continuing education opportunities, and addressing systemic issues such as workload and resource constraints that impact nurse work engagement and retention. The findings of this study underscore the importance of tailored educational interventions for newly hired nurses in Ghana. Nursing schools and healthcare institutions could use these insights to develop orientation programs that address the unique needs and challenges faced by novice nurses.

## Conclusion

By employing qualitative, humanistic techniques this study has shown that newly hired nurses perceive their nursing occupation as emotionally demanding and mentally distressing but worthy and fulfilling. Their job realities do not match their entry expectations and assumptions. The main stressors confronting them on the job include the number of patients, negative patients, and senior nurses’ attitudes toward them. In dealing with the stressors the newly hired nurses rely on their religious faith, family support, and transactional friendships with senior nurses for support. Being nurses further brought them social prestige which, combined with a perceived lack of alternative sources of income, mitigates turnover intentions among them. In the face of these findings, institutional support systems that have specialized components targeted at providing specialized emotional and psychological assistance to newly hired nurses are recommended. These could help mitigate the negative experiences and improve resilience and health for these nurses. Other measures that seek to increase the sense of worth and prestige, and positive interpretations of work roles and work circumstances could help increase nurses’ engagement with the work and resilience. The narratives of the newly hired nurses offer information that can be valuable for nursing trainers in preparing their students for the realities of the job and health institutions in devising ways of supporting newly hired nurses who are at the beginning of their careers.

## Supplemental Material

sj-docx-1-son-10.1177_23779608241279911 - Supplemental material for Exploring Work-Related Experiences of Newly Hired Hospital Nurses in Ghana: A Qualitative Study Supplemental material, sj-docx-1-son-10.1177_23779608241279911 for Exploring Work-Related Experiences of Newly Hired Hospital Nurses in Ghana: A Qualitative Study by Ernest Darkwah, Francis Annor, Seth Oppong and Sylvia Hagan in SAGE Open Nursing

sj-pdf-2-son-10.1177_23779608241279911 - Supplemental material for Exploring Work-Related Experiences of Newly Hired Hospital Nurses in Ghana: A Qualitative Study Supplemental material, sj-pdf-2-son-10.1177_23779608241279911 for Exploring Work-Related Experiences of Newly Hired Hospital Nurses in Ghana: A Qualitative Study by Ernest Darkwah, Francis Annor, Seth Oppong and Sylvia Hagan in SAGE Open Nursing
