# Supplementary material for: Exploring Work-Related Experiences of Newly Hired Hospital Nurses in Ghana: A Qualitative Study
Source: SAGE Open Nurs. 2024 Oct 9;10:23779608241279911. doi: 10.1177/23779608241279911 (PMC11465299; doi:10.1177/23779608241279911)
Supplement: sj-docx-1-son-10.1177_23779608241279911 - Supplemental material for Exploring Work-Related Experiences of Newly Hired Hospital Nurses in Ghana: A Qualitative Study [file sj-docx-1-son-10.1177_23779608241279911.docx]

**INTERVIEW GUIDE**

**Section A: Informed consent form**

Hello, thank you for volunteering to participate in this research. As you may already have been informed this research seeks to explore the work-related lived experiences of newly hired hospital nurses in the Eastern Region of Ghana. The idea is to generate insights into newly hired nurses’ experience on the job and how these experiences influence work, wellbeing and future aspirations. Your participation will be valuable in helping achieve these research objectives. Participation will involve granting an interview in which you will be requested to share your experiences as a newly-hired hospital nurse. The interview will not take more than one (1) hour. Your responses will be kept confidential and anonymous, and you are free to withdraw from the study at any time without any consequences. The information you provide will be used solely for research purposes. Thank you.

Before participating please confirm that your participation is voluntary by signing here:

…………………………….. ………………………………

(Signature) (Date)

**Section B: Demographic information**

1. How old are you?
2. What is your gender?
3. How long have you worked in your current hospital or clinic?
4. How long have you worked as a nurse?
5. What is your current position or specialization?

**Section C: Research questions**

(1): How have you experienced the health care work so far since you joined?, (2) Do your experiences so far match the expectations with which you joined this profession?, (3) What would you say are the main stressors confronting you on this job?, and, (4) Are there any things, persons, factors or aspects of this work and work environment that you would say are resourceful to you and help you cope with the job?.

(1) How have you experienced the health care work so far since you joined?

*How has your perception of healthcare work changed over time?*

(2) Do your experiences so far match the expectations with which you joined this profession?

*In what ways have your experiences met or not met these expectations? Can you provide specific examples of any discrepancies?*

(3) What would you say are the main stressors confronting you on this job?

*Why would you describe them as stressors and how do they stress you?*

(4) Are there any things, persons, factors or aspects of this work and work environment that you would say are resourceful to you and help you cope with the job?.

**Closing** 4b. Before we conclude, do you have any additional comments or suggestions that you would like to share?

If you have any follow-up questions or concerns regarding your participation in this research, please feel free to contact this email address: [edarkwah@ug.edu.gh](mailto:edarkwah@ug.edu.gh) for assistance.

Thank you once again for your time and participation.
